# Bioequivalence and pharmacodynamics of a generic dabigatran etexilate capsule in healthy Chinese subjects under fasting and fed conditions

**DOI:** 10.1002/prp2.593

**Published:** 2020-04-27

**Authors:** Xin Li, Lihua Liu, Bing Xu, Qian Xiang, Yuan Li, Ping Zhang, Yangyang Wang, Qiufen Xie, Yong Mao, Yimin Cui

**Affiliations:** ^1^ Department of Pharmacy The Third Hospital of Changsha Changsha The People’s Republic of China; ^2^ Department of Pharmacy Peking University First Hospital Beijing The People’s Republic of China; ^3^ Chengdu Brilliant Pharmaceutical Co. Ltd Chengdu The People’s Republic of China

**Keywords:** bioequivalence, food effect, generic dabigatran etexilate, pharmacokinetics, pharmacodynamics

## Abstract

To assess bioequivalence of a generic dabigatran etexilate capsule in healthy Chinese subjects under fasting and fed conditions. This was an open‐label, single‐center, randomized four‐period crossover study with a 7‐day washout period. A single oral dose of 150 mg generic dabigatran etexilate capsule (test drug) or a commercial dabigatran etexilate capsule (Pradaxa^®^, reference drug) was given to healthy volunteers under the fasting and fed conditions. Plasma concentrations of total and free dabigatran were detected using a validated HPLC‐MS/MS method. A noncompartmental method was used for pharmacokinetic analysis and established coagulation assays were applied for pharmacodynamic analysis. The 90% CIs of the test/reference ratios of C_max_, AUC_0‐t_, and AUC_0‐∞_ for the total dabigatran concentration were 92.57%‐106.58%, 91.63%‐106.32%, and 92.54%‐106.17%, respectively, under fasting condition, and 99.30%‐110.74%, 98.58%‐105.37%, and 97.75%‐103.99%, respectively, under fed conditions. The 90% CIs of the ratios of the parameters for the free dabigatran were 93.18%‐106.98%, 92.13%‐107.10%, 92.89%‐106.48%, respectively, under fasting condition, and 100.05%‐110.89%, 99.37%‐106.23%, 97.59%‐103.98%, respectively, under the fed condition. Additionally, the upper limit of the 90% CIs for σWT/σWR was below 2.5. There were no significant differences in the coagulation parameters including thrombin clotting time, activated partial thromboplastin time, and anti‐IIa activity between the two preparations. The generic dabigatran etexilate capsule is bioequivalent to the brand‐named product in healthy Chinese volunteers under fasting and fed conditions. The two products have comparable pharmacodynamic parameters, with a good safety profile. In addition, food intake influences absorption of both products in a similar way.

## 1 INTRODUCTION

Atrial fibrillation (AF) is one of the most prevalent arrhythmia, and it is an independent hazard index for ischemic stroke.^1^ The global prevalence of AF adjusted by age was estimated to be 596 and 373 per 100 000 in males and females.^2^ Due to population ageing, the age‐adjusted prevalence (≥30 years) of AF in China is rising rapidly, reaching to about 0.65%, and 9 million individuals aged ≥60 years are estimated to have AF by 2050.^3^ Risk‐based anticoagulant therapy is recommended to reducing thromboembolism risk in AF patients.^1^, ^4^ Although oral administration of warfarin was proved to reduce stroke incidence in non‐valvular AF (NVAF), it presents several drawbacks including long response time, complex drug interactions, and requirement for frequent monitoring. Thus, novel anticoagulants that overcome the drawbacks are needed.

Over the recent years, the treatment of NVAF has advanced rapidly, and several novel oral anticoagulants (NOACs), including dabigatran etexilate, have already applied in clinical practice.^5^ As a new oral reversible thrombin inhibitor, dabigatran etexilate is rapidly absorbed and biotransformed into an active form of dabigatran, which directly inhibits thrombin (factor IIa) activity and production.^5^ The US Food and Drug Administration (FDA) approved a brand‐named product of dabigatran etexilate (Pradaxa^®^, Boehringer Ingelheim International GmbH, Ingelheim am Rhein, Germany) for reducing the risk of stroke and systemic embolism in patients with NVAF in 2010, which was launched in China in 2013.^6^, ^7^ ^.^The 2019 AHA/ACC/HRS guidelines recommend using NOACs including dabigatran etexilate over warfarin in NOAC‐eligible AF patients except those with a mechanical heart valve or moderate‐to‐severe mitral stenosis.^4^ However, the clinical application of dabigatran etexilate, mainly Pradaxa^®^, in Chinese NVAF patients was not satisfactory; the discontinuation rate of dabigatran therapy was higher compared with warfarin, due to its high price that many patients cannot afford.^8^ Thus, research and development of generic products of dabigatran etexilate to remove the barrier that the brand‐named product of dabigatran etexilate posed and provide the availability and accessibility to dabigatran therapy to Chinese NVAF patients is urgently needed.

Currently, a generic product of dabigatran etexilate (150‐mg capsule) is being investigated and developed by Chengdu Brilliant Pharmaceutical Co., Ltd., in China. In order to meet the requirements by the Chinese regulatory authorities for marketing authorization for generic products in China, the present study was carried out to assess bioequivalence (BE) of this generic dabigatran etexilate capsule, with reference to the brand‐named product (Pradaxa^®^), in healthy Chinese subjects under fasting and fed conditions. In addition, the pharmacodynamics and safety of this generic product were also assessed.

## 2 MATERIALS AND METHODS

### 2.1 Study population

A total of 92 healthy subjects aged 18‐65 years, body mass index 19‐26 kg/m^2^ were recruited in this study. During screening visits, all the volunteers were assessed for physical examinations, 12‐lead electrocardiogram, laboratory tests (hematology, urinalysis, blood chemistry, and coagulation function), serologic tests (hepatitis B surface antigen, hepatitis C virus antibody, human immunodeficiency virus antibody, and syphilis antibody), vital signs, and past medical history. Participants agreed to use effective methods of contraception from 2 weeks before screening to 3 months after the end of the trial.

Subjects were excluded from the study if they had (a) abnormal physical examinations, vital signs, electrocardiogram, or laboratory results of clinical significance based on the physician's judgment; (b) any history and/or presence of cardiovascular, endocrine, neurological, gastrointestinal, respiratory, hematological, psychiatric, and hepatic and renal diseases; (c) allergy to any food or drugs; (d) pregnancy or lactation; (e) surgery performed 1 month before this study or planned during this study; (f) blood loss ≥200 mL, blood donation, and/or involvement in any other clinical studies 3 months before this study; (g) heavy consumption of coffee, tea, caffeinated beverage, or alcohol abusers ≥14 units alcohol per week; (h) heavy smokers ≥5 cigarettes per day; (i) drug abusers and positive drug abuse screening test (morphine, tetrahydrocannabinol, methamphetamine, 3,4‐methylenedioxyamphetamine, cocaine, and ketamine); (j) treatment of any drugs within the 14 days, or any drugs that affect blood coagulation (namely acetylsalicylic acid, non‐steroidal anti‐inflammatory drugs, coumarin, etc) within the 30 days before the first administration of the study drug.

### 2.2 Study drugs

The test medicinal product was a generic dabigatran etexilate capsule 150 mg produced by Chengdu Brilliant Pharmaceutical Co., Ltd. (batch number: 011803, expiration date March 2021). The reference formulation was the brand‐named dabigatran etexilate capsule 150 mg (Pradaxa^®^) manufactured by Boehringer Ingelheim International GmbH (batch number: 604199, expiration date April 2019). Both the generic and brand‐named dabigatran etexilate capsules were supplied by Chengdu Brilliant Pharmaceutical Co., Ltd.

### 2.3 Study design

This clinical trial strictly adheres to the ethical guidelines for human medical research of The Declaration of Helsinki. The protocol of this study and the informed consent were approved by the Ethics Committee of the Third Hospital of Changsha (Approval No. 2018EC‐024). Before the trial, the investigators explained the purpose, procedures, and possible risk of the trial and the principle of voluntary participation to all subjects. Written informed consent was provided by each volunteer before the study. This study was registered at http://www.chinadrugtrials.org.cn (Identifier: ChiCTR1800019511) and Center for Drug Evaluation NMPA (Identifier: 201800202‐01).

The study was performed in two separate cohorts of healthy volunteers under fasting and fed conditions, respectively. An open‐label, single‐center, randomized single‐dose, four‐period crossover design was used for both cohorts. Eligible healthy volunteers were recruited and randomly divided into either a test‐reference‐test‐reference sequence group or a reference‐test‐reference‐test sequence group according to the number table obtained by the Statistical Analysis System (SAS) 9.1 software, and each period was separated by a 7‐day washout period.

In the fasting cohort, all the subjects were hospitalized 1 day before the study and underwent an over‐night fasting for at least 10 hours. The next morning, each subject orally administered a single dose (150‐mg capsule) of either the test or reference dabigatran etexilate with 240 mL warm water, and was restricted from drinking for 1 hour before drug administration and 2 hours after administration. For the periods 2‐4, underwent the pre‐specified treatment sequence and also followed the above procedures.

In the fed cohort, after hospitalization and fasting over‐night for at least 10 hours, the subjects were fed with a standard high‐fat meal, which contained 800‐1000 kcal (500‐600 kcal fat, 150 kcal protein, and 250 kcal carbohydrates), 30 minutes before administration of the study drug. Other study procedures were similar with that in the fasting cohort.

### 2.4 Pharmacokinetic blood sampling and analytical method

Blood samples were collected to the EDTA‐K_2_ vacuum tube (containing 91.2 mM ammonium chloride) by inserting a cannula into the forearm vein prior to administration. In the fasting cohort, 4 mL venous blood was collected at 0, 15, 30, 45, 60, 80, and 100 min and 2.0, 2.5, 3.0, 3.5, 4.0, 5.0, 6.0, 8.0, 10.0, 12.0, 24.0, and 48.0 hours after dosing during each of the four periods to determine the plasma concentrations of total and free dabigatran. In the fed cohort, 4 mL venous blood (containing 91.2 mM ammonium chloride) was collected at 0, 0.5, 1.0, 1.5, 2.0, 2.5, 3.0, 3.5, 4.0, 4.5, 5.0, 5.5, 6.0, 8.0, 10.0, 12.0, 24.0, and 48.0 hours after dosing during each of the four periods. The blood samples were centrifuged at 1700 g and 4°C for 10 minutes. Plasma was collected into two tubes (one for determination of total dabigatran, and the other containing 0.33% formic acid and 166.7 mM ammonium formate for free dabigatran), and stored at −80°C until testing.

The plasma concentrations of dabigatran etexilate were determined by a home‐validated liquid chromatography‐tandem mass spectrometry (LC‐MS/MS) method. Dabigatran etexilate and dabigatran etexilate‐^13^C_6_ were purchased from TLC PharmaChem Inc (Ontario, Canada) with purities of 98.7% and 99.0%, respectively. The sample separation was performed on a GL Sciences‐AQ‐C_18_ column (2.1 mm × 150 mm, 3 µm, GL Sciences, Japan) connected with a C_18_ guard column (Guard Cartridge System, USA) at 35°C. Two mobile phases were mobile phase A of 0.1% formic acid containing 15.0 mM ammonium formate in water and mobile phase B of acetonitrile and water (0.1% formic acid containing 150 mM ammonium formate; 9:1, v/v).The flow rate was set at 0.4 mL/min. The gradient elution was set as follows: 45% solvent B (0‐1 minutes), 45% solvent B (1‐2.3 minutes), 95% solvent B (2.31‐2.8 minutes), and 10% solvent B (2.81‐4.5 minutes).

Positive‐mode electrospray ionization was selected for mass spectral analysis, and *m/z* 472.3 → 289.3 and *m/z* 478.5 → 295.2 were chosen, respectively, for dabigatran etexilate and internal standard (IS) in the multiple reaction monitoring mode. The electrospray voltage was set at 4000 V, the declustering potential was set to 85V, and the collision energy used was 40 V for dabigatran etexilate. Analyst® software 1.6.1 was used for MS parameters optimization, data acquisition, and data processing.

The standard calibration curves with good linearity were built for both total and free dabigatran within the concentration range of 1.0‐300.0 ng/mL. The precision (% CV) was within 6.5%‐9.3% for total dabigatran and 2.5%‐9.8% for free dabigatran, respectively. The intra‐batch accuracy was between 92.1% and 103.1% for total dabigatran and 101.1% and 104.7% for free dabigatran, respectively. The inter‐batch accuracy was between 95.7% and 103.5% for total dabigatran and 95.6% and 104.0% for free dabigatran, respectively. The lower limit of quantitation (LOQ) of both total and free dabigatran in the plasma was 1.000 ng/mL. The utilized methods, as indicated by the validation, were suitable for large amounts of biomedical samples.

### 2.5 Pharmacodynamic blood sampling and analysis

The pharmacodynamic (PD) effects of dabigatran etexilate were assessed by measurement of anti‐IIa activity, thrombin clotting time (TT), and activated partial thromboplastin time (aPTT). Blood samples were collected at 0, 2 8, and 12 hours after dosing during any one of the four periods for test or reference product. The blood samples were centrifuged at 2500 g for 10 minutes. Plasma was collected and stored at −80°C until testing.

The analyses of aPTT and TT were performed by validated clotting assays with Sysmex CS2000i automatic coagulation analyzer (Wakinohama‐Kaigandori, Chuo‐ku, Kobe, Japan). Anti‐IIa activity was determined by a chromogenic substrate method as previously described.^9^

### 2.6 Safety

The safety of the test and reference dabigatran etexilate capsules was assessed by vital signs monitoring, physical examination, laboratory tests (hematology, biochemistry, and urinalysis), and electrocardiogram and adverse events (AEs) during the study. Abnormalities that were considered clinically significant by the investigators after randomization were recorded as adverse events.

### 2.7 Statistical analysis

The C_max_, AUC_0‐t_, and AUC_0‐∞_ of total and free dabigatran were the main observed pharmacokinetic parameters, and the T_max_ of total and free dabigatran was secondary pharmacokinetic parameter. Following logarithmic conversion, analysis of variance (ANOVA) on the main pharmacokinetic parameters was done to estimate the ratio of the test drug to the reference drug and its 90% confidence interval (CI). Statistical analysis was performed by parametric mixed‐model accounting for subjects as random effect and period, sequence, and formulation as fixed effect. T_max_ difference between the two products was assessed by nonparametric Wilcoxon test. Two one‐sided t tests were conducted to determine the bioequivalence. Referring to the Draft Guidance on Dabigatran Etexilate Mesylate issued by the FDA,^10^ bioequivalence was demonstrated between the test and reference preparations if the 90% CI of C_max_, AUC_0‐t_, and AUC_0‐∞_ fell within 80.00%‐125.00% and the upper limit of the 90% CI for the test‐to‐reference ratio of the within‐subject variability was less than 2.5. PD analysis was also performed by ANOVA using a standard 2 × 2 crossover design. Pearson correlation test was used to find relationship between the total dabigatran concentration and PD parameters. Noncompartmental pharmacokinetic analysis was done by WinNonlin software version 7.0 (Pharsight Corporation, California, USA), and other statistical analysis was analyzed with SAS software version 9.4.

## 3 RESULTS

### 3.1 Demographics and baseline characteristics

A total of eligible 92 subjects were recruited, 46 in the fasting cohort, and 46 in the fed cohort. The baseline characteristics, including demographics, height, weight, and body mass index (BMI), are shown in Table 1. Only one subject in the fasting cohort withdrew from the study in the third period 4 hours after dosing of the test product, and the other subjects completed all the periods and were included in the final BE analysis.

**Table 1:** Baseline characteristics of the subjects in the fasting and fed cohorts

Demographics	Fasting (n = 46)	Fed (n = 46)
Male/female (N)	33/13	34/12
Age (years)	23.5 ± 5.3 (18‐39)	25.2 ± 6.4 (18‐41)
Height (cm)	165.8 ± 7. 6 (148.5‐180.0)	164.7 ± 6.7 (146.0‐177.0)
Weight (kg)	61.5 ± 5.7 (50.5‐73.8)	60.2 ± 5.1 (51.2‐71.4)
BMI (kg/m^2^)	22.4 ± 1.6 (19.9‐25.6)	22.2 ± 1.6 (19.7‐25.8)

Abbreviation: BMI, body mass index.

Data on age, height, weight, and BMI are expressed as mean ± standard deviation.

### 3.2 Pharmacokinetics

**Figure 1:**
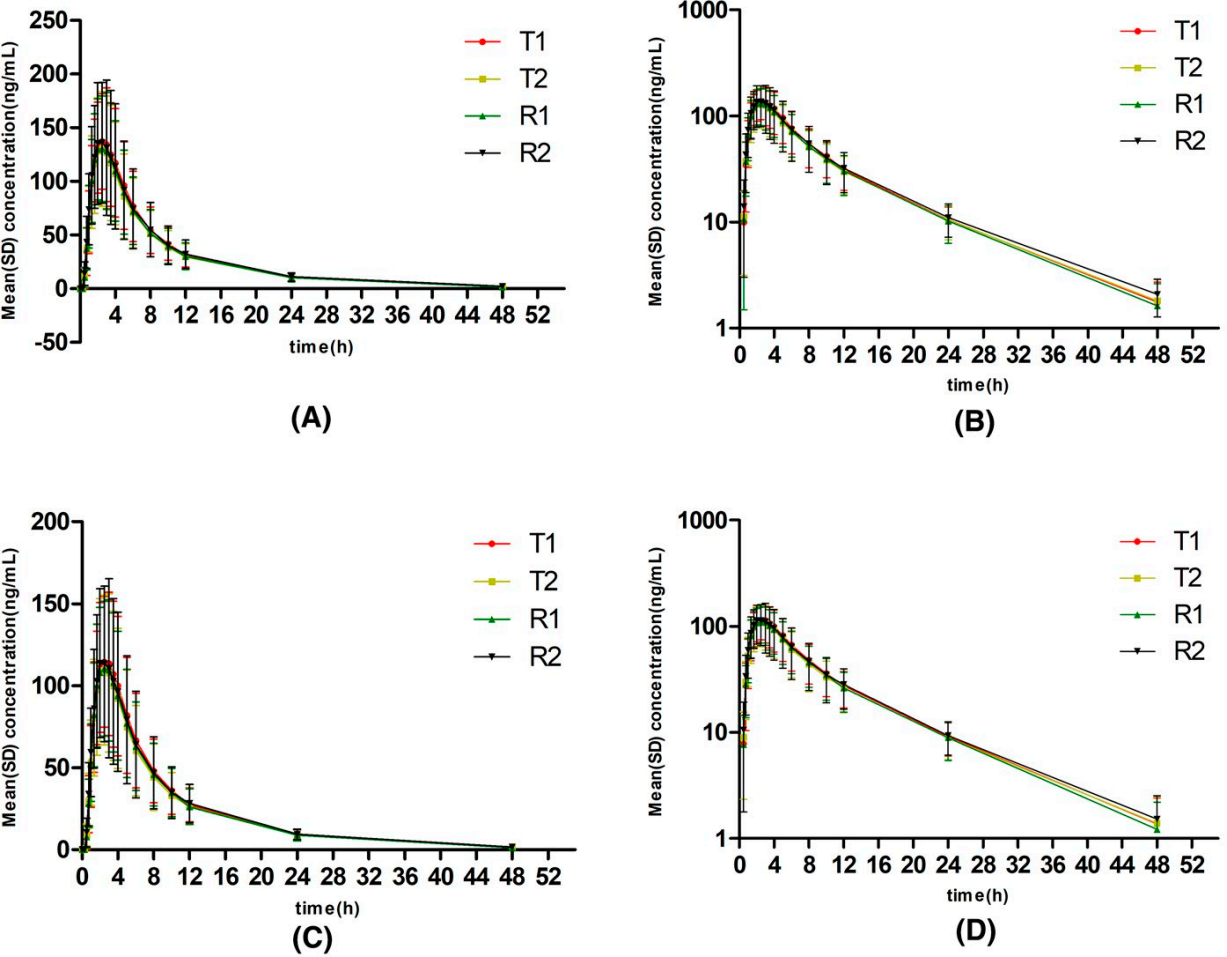
Plasma concentration‐time profile of single dose of test and reference dabigatran etexilate capsules in 45 healthy subjects under the fasting condition. (A), plasma concentration‐time profile for total plasma concentration; (B) semi‐logarithmic plasma concentration‐time profile for total plasma concentration; (C) plasma concentration‐time profile for free plasma concentration; and (D) semi‐logarithmic plasma concentration‐time profile for free plasma concentration

**Figure 2:**
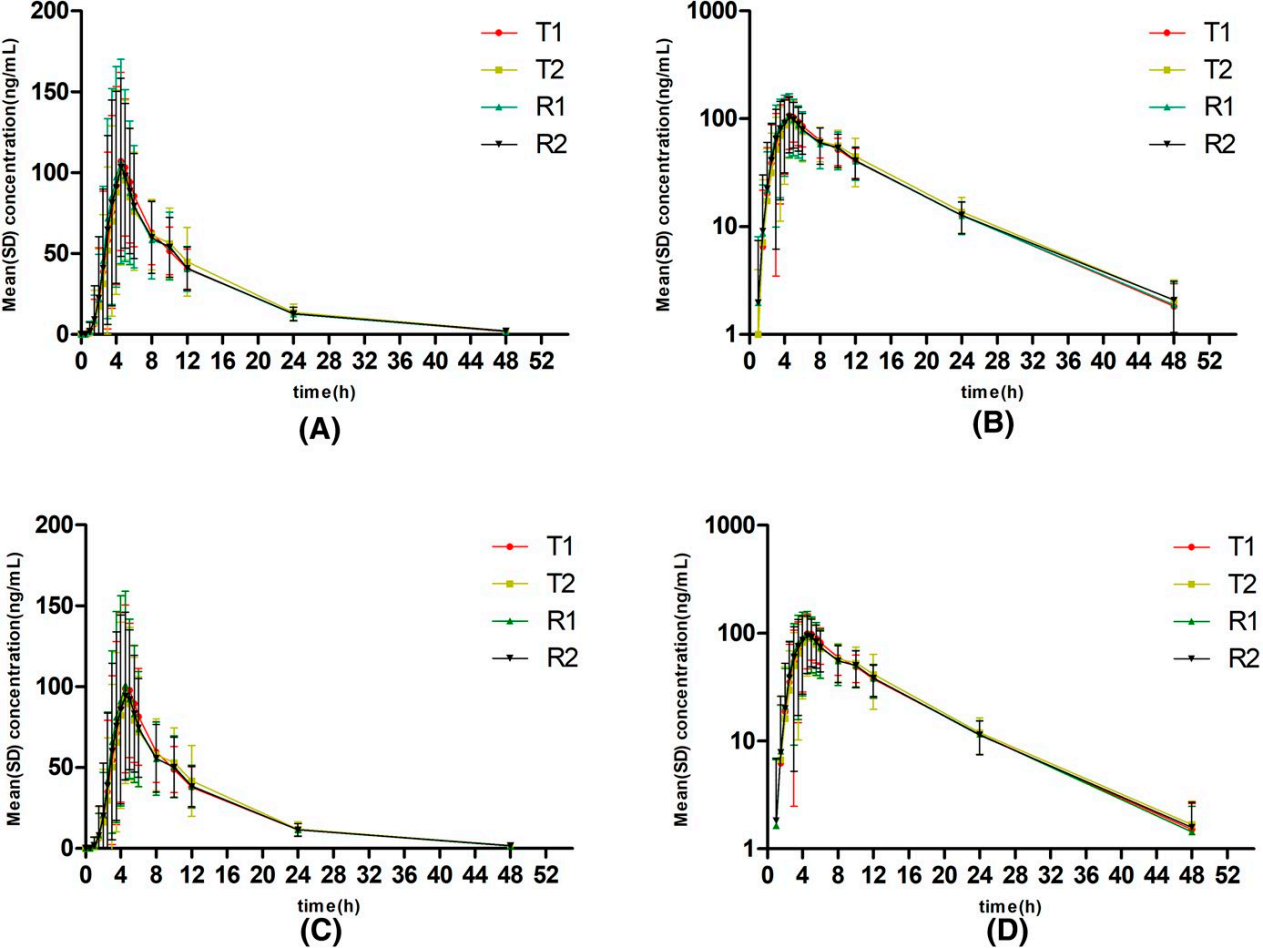
Plasma concentration‐time profile of single dose of test and reference dabigatran etexilate capsule in 46 healthy subjects under the fed condition. (A), plasma concentration‐time profile for total plasma concentration; (B) semi‐logarithmic plasma concentration‐time profile for total plasma concentration; (C) plasma concentration‐time profile for free plasma concentration; and (D) semi‐logarithmic plasma concentration‐time profile for free plasma concentration

The plasma concentration‐time profile of total and free dabigatran after a single dose of the test and reference dabigatran etexilate under the fasting and fed conditions are shown in Figures 1 and 2, respectively, and the main pharmacokinetic data of total and free dabigatran and the BE analysis are summarized in Tables 2 and 3, respectively.

**Table 2:** Pharmacokinetic parameters after a single oral dose of 150 mg test and reference dabigatran etexilate capsules in healthy Chinese subjects under fasting and fed conditions

	Total dabigatran		Free dabigatran	
Parameter (unit)	Test	Reference	Test	Reference
Fasting (n)	91^a^	91^a^	91^a^	91^a^
T_max_ (h)	2.00 (1.33, 5)	2.00 (1.33, 4)	2.00 (1.33, 5)	2.00(1.33, 4)
C_max_ (ng/mL)	144.85 ± 52.73	147.22 ± 56.77	121.91 ± 45.31	123.28 ± 48.07
AUC_0‐t_ (h·ng/mL)	1227.94 ± 457.93	1247.83 ± 485.03	1041.63 ± 415.79	1050.18 ± 427.61
AUC_0‐∞_ (h·ng/mL)	1261.30 ± 454.44	1277.67 ± 485.00	1079.54 ± 408.04	1087.48 ± 420.08
T_1/2z_ (h)	8.92 ± 1.26	8.90 ± 1.28	8.68 ± 1.49	8.67 ± 1.64
Fed (n）	92	92	92	92
T_max_ (h)	4.50 (2, 12)*	4.50 (2.5, 12)*	4.50 (2, 12)*	4.50 (2.5, 12)*
C_max_ (ng/mL)	127.59 ± 44.37	123.70 ± 48.72	120.73 ± 42.17	116.26 ± 45.68
AUC_0‐t_ (h·ng/mL)	1176.09 ± 347.33	1156.91 ± 357.35	1080.94 ± 337.39	1056.57 ± 349.00
AUC_0‐∞_ (h·ng/mL)	1207.82 ± 350.61	1197.13 ± 357.73	1110.97 ± 332.43	1108.28 ± 341.95
T_1/2z_ (h)	8.14 ± 1.14	8.21 ± 1.11	7.89 ± 1.22	7.89 ± 1.20

Abbreviations: AUC_0–∞_, area under the curve extrapolated to infinity; AUC_0–t_, area under the curve to the last measurable concentration; C_max_, maximum plasma concentration; h, hours; T_1/2z_, elimination half‐life; T_max_, time to C_max_.

Data are expresses as arithmetic mean ± standard deviation; T_max_, the values are expressed in terms of median (range).

^a^ One subject in the fasting cohort withdrew from the study in the third period 4 h after dosing of the test product, and thus, there was no measurement for the third (test product) and fourth (reference product) periods.

* *P* < .05, compared with fasting condition.

**Table 3:** Bioequivalence between the test (T) and reference (R) dabigatran etexilate capsules in healthy Chinese subjects under fasting and fed conditions

Parameter (unit)	Total dabigatran				Free dabigatran			
	T/R GMR (%)	90% CI (%)	σ_WT_/σ_WR_	90% CI of σ_WT_/σ_WR_	T/R GMR (%)	90% CI (%)	σ_WT_/σ_WR_	90%CI of σ_WT_/σ_WR_
Fasting								
C_max_ (ng/mL)	99.33	92.57‐106.58	0.82	0.64‐1.06	99.84	93.18‐106.98	0.77	0.60‐0.99
AUC_0‐t_ (h·ng/mL)	98.70	91.63‐106.32	0.81	0.63‐1.05	99.33	92.13‐107.10	0.84	0.65‐1.08
AUC_0‐∞_ (h·ng/mL)	99.12	92.54‐106.17	0.82	0.64‐1.06	99.46	92.89‐106.48	0.83	0.64‐1.07
Fed								
C_max_ (ng/mL)	104.86	99.30‐110.74	0.90	0.70‐1.15	105.33	100.05‐110.89	0.82	0.63‐1.05
AUC_0‐t_ (h·ng/mL)	101.91	98.58‐105.37	0.99	0.77‐1.28	102.74	99.37‐106.23	0.90	0.70‐1.16
AUC_0‐∞_ (h·ng/mL)	101.34	97.75‐103.99	0.98	0.76‐1.26	101.26	97.59‐103.98	0.88	0.68‐1.15

Abbreviations: AUC_0–∞_, area under the curve extrapolated to infinity; AUC_0–t_, area under the curve to the last measurable concentration; C_max_, maximum plasma concentration; GMR, geometric mean ratio; σ_WR_, intra‐individual variability of reference product; σ_WT_, intra‐individual variability of test product.

As shown in Table 3, the geometric mean ratios (90% CI) of the test‐to‐reference products for C_max_, AUC_0‐t_, and AUC_0‐∞_ for both total and free dabigatran were all within 80.00%‐125.00%. In addition, the upper limit of the 90% CI for the test‐to‐reference ratio of the within‐subject variability (σ_WT_/σ_WR_) was all less than 2.5 when under the fasting and fed conditions. The differences between the test capsule and reference capsule in the T_max_ and elimination half‐life (T_1/2_) values were not statistically significant in both fasting and fed conditions (Table 2). Based on the FDA guidance,^10^ the test dabigatran etexilate capsule was considered bioequivalent to the reference dabigatran etexilate capsule.

As shown in Table 2, food intake seemed to have an influence on the T_max_ of dabigatran. The T_max_ of total and free dabigatran was reached 2 hours after administration in the fasting condition, but 4.5 hours in the fed condition (Table 2, *P* < .05).

### 3.3 Pharmacodynamics

**Figure 3:**
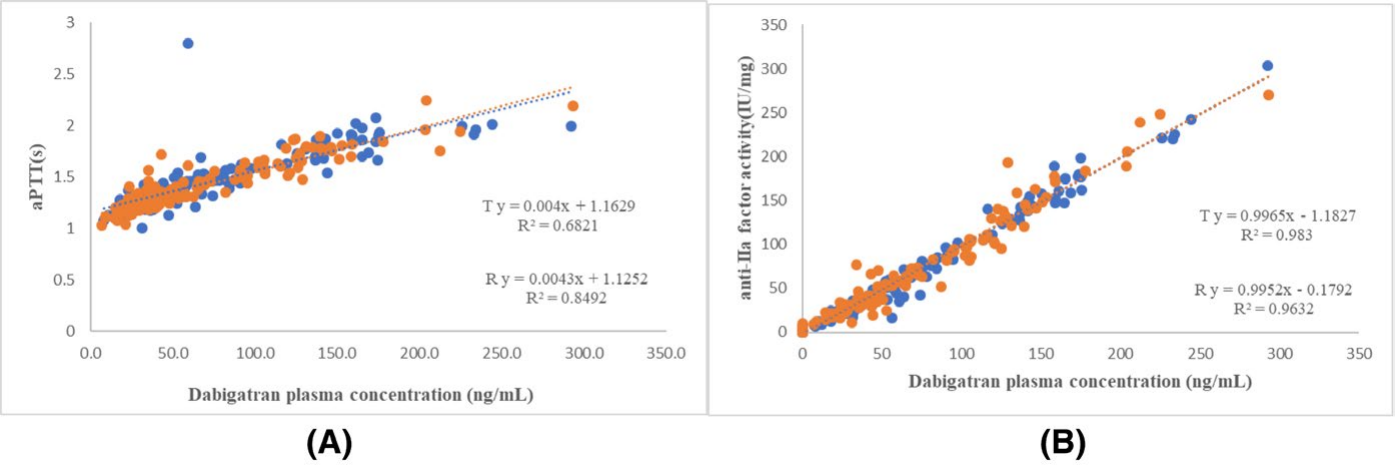
Dabigatran plasma levels *versus* laboratory coagulation results under the fasting condition. (A) aPTT; and (B) anti‐IIa activity

**Figure 4:**
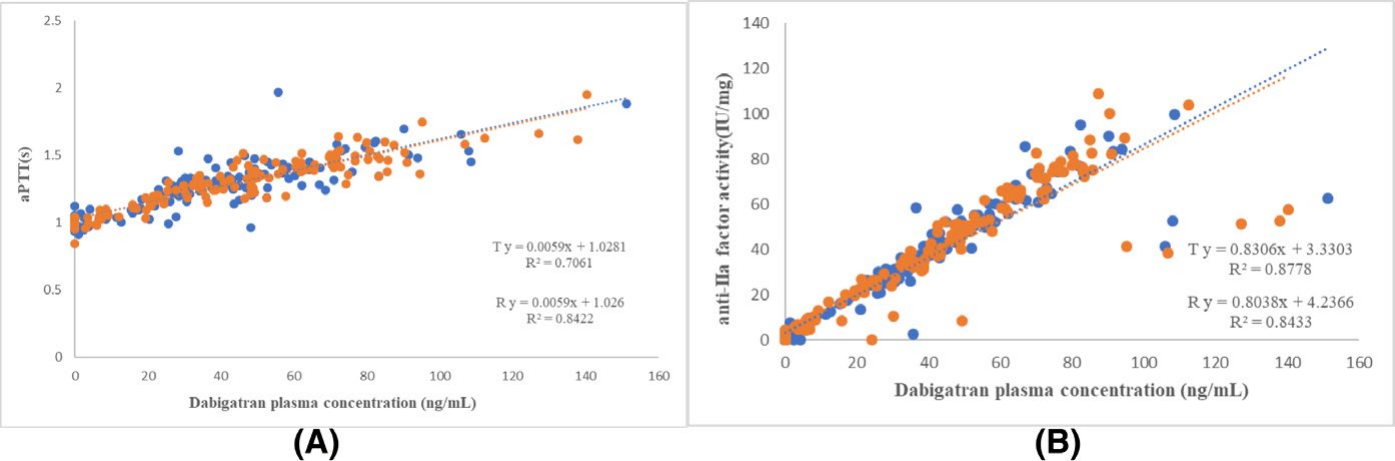
Dabigatran plasma levels versus laboratory coagulation results under the fed condition. (A) aPTT; and (B) anti‐IIa activity

Anti‐IIa activity after a single oral dose of dabigatran etexilate capsules under fasting and fed conditions was shown in Table 4. The geometric mean ratios of area under efficacy curve (AUEC) of aPTT, TT, and anti‐IIa activity were shown in Table 5. The aPTT, TT, and anti‐IIa activity were not significantly different between the two products (all *P* > .05). As shown in Figures 3 and 4, a linear correlation was found between plasma concentrations of total dabigatran and coagulation parameters (ie, aPTT and anti‐IIa activity) for both test and reference capsule products under the fasting and fed conditions (*P* < .05).

**Table 4:** Anti‐IIa activity after a single oral dose of 150 mg test (T) and reference (R) dabigatran etexilate capsules in healthy Chinese subjects under fasting and fed conditions

Time	Anti‐IIa activity of T (ng/mL)	Anti‐IIa activity of R (ng/mL)
Fasting		
0h	1.83 ± 1.86	1.73 ± 1.96
2h	133.21 ± 62.79	125.76 ± 53.58
8h	55.39 ± 25.93	49.95 ± 20.81
12h	31.81 ± 13.65	26.76 ± 10.96
Fed		
0h	0.79 ± 0.93	1.18 ± 1.12
2h	17.26 ± 23.61	21.41 ± 28.34
8h	54.57 ± 20.74	59.14 ± 19.86
12h	35.73 ± 11.36	43.56 ± 15.37

**Table 5:** Pharmacodynamic analysis after a single oral dose of 150 mg test (T) and reference (R) dabigatran etexilate capsules in healthy Chinese subjects under fasting and fed conditions

Parameters	T/R GMR of AUEC (%)	95% CI (%)
Fasting		
aPTT	99.80	90.52‐110.03
TT	106.75	88.03‐129.46
Anti‐IIa activity	91.34	78.45‐106.34
Fed		
aPTT	85.93	73.60‐100.34
TT	102.53	86.37‐121.72
Anti‐IIa activity	97.66	85.56‐111.48

Abbreviations: aPTT, activated partial thromboplastin time; AUEC, area under efficacy curve; GMR, geometric mean ratio; TT, thrombin clotting time.

TT values were beyond the detectable limit in 54 measurements (30 when taking the reference product and 24 taking the test product) in the fasting cohort, and four measurements (two when taking reference product and two taking test product) in the fed cohort. When these values were considered as missing values, TT was correlated with plasma total dabigatran concentration (*P* < .05), with *R* ^2^ values of 0.84 and 0.80 for the test product, and 0.80 and 0.70 for the reference product, respectively, under the fasting and fed conditions.

### 3.4 Safety

In the fasting cohort, 10 subjects (10/46, 21.7%) experienced 13 AEs, of which 11 AEs were considered possibly related to the drug administration; five and six were likely related to the test and reference products, respectively. All AEs were mild (Grade I), with nasal hemorrhage (n = 3) and abdominal pain (n = 3) being the most frequent. At the last scheduled visit, 12 AEs were completely recovered and one improved. In the fed cohort, nine subjects (9/46, 19.6%) experienced a total of 12 AEs, of which eight were possibly related to the study drugs; five occurred in the test product period and three in the reference product period. All AEs were mild (Grade I), with diarrhea (n = 2) and hyperbilirubinemia (n = 2) being the most frequent. At the last scheduled visit, 11 AEs were recovered and one improved. There were no serious AEs or AEs leading to withdrawal in the fasting and fed cohorts. The difference in the incidence of AEs between the test and reference products was not statistically significant.

## 4 DISCUSSION AND CONCLUSIONS

In the present study, all PK parameters, including C_max_, AUC_0‐t_, and AUC_0‐∞_, were similar between the test and reference products in healthy Chinese subjects under both fasting and fed conditions. Moreover, the two products also had similar PD profiles, in terms of aPTT, TT, and anti‐IIa activity. In addition, mild and self‐limited AEs occurred in only approximately 20% of subjects after administration of the test or reference product. These findings indicate that the generic dabigatran etexilate capsule is bioequivalent to the brand‐named Pradaxa^®^ capsule, and the two products have comparable PD parameters with a good safe profile. To the best of our knowledge, this is the first study that determines BE of a generic dabigatran etexilate capsule, as well as its PD and safety in healthy Chinese volunteers, with reference to a brand‐named dabigatran etexilate capsule.

There was no significant difference in the main PK parameters (ie, C_max_, AUC_0→t_, and AUC_0→∞_) between two dabigatran etexilate capsules under either fasting or fed condition in the present study. The test‐to‐reference ratio of geometric mean ratios (90% CI) for the C_max_, AUC_0‐t_, and AUC_0‐∞_ for both total and free dabigatran were within 80.00%‐125.00% and the upper limit of 90% CI for σ_WT_/σ_WR_ was less than 2.5 under fasting and fed conditions. These findings are within the conventional acceptance for bioequivalence evaluation. These main PK parameters of total dabigatran are compared with those data from previous studies.^11^, ^12^, ^13^, ^14^ The T_max_ obtained in the present study is consistent with that reported in the Caucasian^11^, ^13^ and Japanese subjects.^14^ However, the C_max_ and AUC_0–∞_ of total dabigatran for both products under the fasting and fed conditions in the present study were approximately 20% higher than that in Caucasian subjects, but similar to that in Japanese.^12^ The differences in C_max_ and AUC values between the Chinese and Caucasian may be attributed to several factors such as body weight and serum creatinine,^15^ and more importantly the genetic variants.^16^ Indeed, it has been demonstrated that there is a gene‐dose effect between *ABCB1* and *CES1* genes and peak concentrations of dabigatran etexilate.^15^

In the present study, we investigated the main PK parameters of test and reference preparations under the fed and fasting conditions. Administration with high‐fat meals prolonged the median T_max_ of total and free dabigatran from 2 to 4.5 hours, but did not alter the AUC, indicating that food intake may reduce the rate of absorption without impacting the extent of absorption or drug elimination.

The present study revealed that the main coagulation parameters, aPTT, TT, and anti‐IIa activity, were comparable between the two products by ANOVA analysis. However, the 95% CIs of geometric mean ratios of some measured coagulation parameters fell outside 80.00%‐125.00%, which may be related to the sample size. In this preliminary exploration pharmacodynamic trial, we measured coagulation parameters only in two periods (one R period and one T period). A four‐period trial is expected to more accurately assess the pharmacodynamic equivalence of dabigatran, a highly variable drug. We also found that the aPTT level was correlated with dabigatran concentration although the R^2^ value was relatively low compared with anti‐IIa activity. This finding is consistent with previous observation that there was no change in the aPTT level during dabigatran therapy.^17^ In the present study, TT values exceeded the detectable limit in many subjects, especially in the fasting cohort, which led to missing data in these subjects. It has previously been shown that the dilute thrombin time (dTT) accurately identifies therapeutic and supratherapeutic dabigatran levels, possible due to the fact that dTT has better correlation with dabigatran PK parameters than TT.^17^ Thus, we should use dTT, instead of TT, in our future studies assessing PD. Moreover, in the present study, anti‐IIa activity had a closer correlation with total dabigatran concentration, compared to aPTT and TT, which indicates that it is a potential indicator for clinical monitoring.

In the present study, mild AEs occurred in less than 22% of the subjects under both fasting and fed conditions for the test and reference products, without serious or unexpected AEs observed. Most of the AEs were completely recovered at the final visit. These findings indicate that both the test and reference dabigatran etexilate 150‐mg capsules are safe in healthy Chinese volunteers after a single dose.

In conclusion, the generic dabigatran etexilate capsule is bioequivalent to the brand‐named dabigatran etexilate (Pradaxa^®^) capsule in healthy Chinese subjects under both fasting and fed conditions. The two products have comparable PD parameters with a good safe profile. In addition, food intake influences the rate of absorption for both products.

## 5 ETHIC STATEMENT

This clinical trial strictly adheres to the ethical guidelines for human medical research of The Declaration of Helsinki. The protocol of this study was approved by the Ethics Committee of the Third Hospital of Changsha (Approval No. 2018EC‐024).

## CONFLICT OF INTEREST

All the authors declare no conflicts of interest.

## Data Availability

The datasets used and analyzed during the current study are available from the corresponding author on reasonable request.
